# Can Cessation of Caregiving for Parents Relieve Family Caregivers’ Psychological Distress? A Longitudinal Study Using 17-wave Nationwide Survey Data in Japan

**DOI:** 10.2188/jea.JE20240190

**Published:** 2025-04-05

**Authors:** Takashi Oshio, Ruru Ping

**Affiliations:** 1 Institute of Economic Research, Hitotsubashi University, Tokyo, Japan; 2 Hitotsubashi Institute for Advanced Study, Hitotsubashi University, Tokyo, Japan

**Keywords:** caregiver, informal caregiving, psychological distress, social activity

## Abstract

**Background:**

Informal caregiving of older parents adversely affects the mental health of family caregivers. However, the psychological effects of caregiving cessation and the trajectories of these effects have rarely been examined in Japan. This study addresses this gap.

**Methods:**

Based on a 17-wave nationwide population-based survey in Japan, we analyzed longitudinal data from 8,280 individuals aged 50–59 years in 2005 who started caring for their older parents in 2006 or later and ceased caregiving by 2021. We identified the timings of caregiving onset and cessation and examined the trajectory of psychological distress (PD), defined as a Kessler score ≥5 on the 6-item Kessler scale (range 0–24). Linear mixed models were used to assess the trajectory of PD that evolved after caregiving cessation over the subsequent 3 years.

**Results:**

After adjusting for covariates, the probability of PD decreased by 5.6 percentage points (from 40.8%; 95% confidence interval [CI], 4.1–7.0%) for female caregivers and by 1.9 percentage points (from 31.7%; 95% CI, 0.3–3.5%) for male caregivers at caregiving cessation, remaining stable in subsequent years. For women, higher PD risks related to co-residence with a care recipient diminished quickly upon cessation of caregiving, while the unfavorable impacts of no social activity, extended duration of care, and long-hour daily care persisted in subsequent years. For male caregivers, the impact was generally more limited.

**Conclusion:**

These results suggest that changes in mental health following caregiving cessation warrant serious consideration when developing support programs for former family caregivers.

## INTRODUCTION

Informal caregiving is a well-documented risk factor for mental disorders in middle-aged and older adults. Numerous studies have established a correlation between the psychological burden of caregiving and its detrimental effects on mental health.^1^^–^^7^ Recently research has increasingly focused on the transition from active caregiving to the post-caregiving phase, examining the effects of caregiving cessation on the mental health of former caregivers.^8^^,^^9^

Empirical evidence concerning the mental health trajectories of former caregivers is inconsistent and supports two contrasting hypotheses.^8^^,^^9^ The first hypothesis, grounded in the stress and coping theory,^10^ posits that individuals who endure prolonged and significant strain during caregiving may have the ability to cope with or readjust to overexertion. This, however, can deplete their physical and psychological resources, likely to lead to severe psychological problems after caregiving cessation.^8^^,^^11^^–^^14^ The second hypothesis postulates that the cessation of caregiving responsibilities liberates former caregivers from intensive care burdens and the associated psychological stress, thereby providing relief and leading to positive outcomes.^15^^–^^21^ Both hypotheses have garnered empirical support.^8^^,^^11^^–^^21^ Furthermore, the mental health of former caregivers has been observed to change over time,^13^^–^^16^^,^^19^ and the exit from caregiving roles is often concurrent with the bereavement of a care recipient, where grief tends to interact with the relief from the caregiving burden.^8^^,^^9^^,^^11^^,^^16^^–^^21^

These observations highlight the need for a more rigorous examination of how pre-cessation conditions surrounding caregivers and care recipients affect mental health trajectories.^22^^,^^23^ Factors, such as the intensity and duration of caregiving^18^^,^^21^ and social activity and support,^11^^,^^13^^,^^17^ have been identified as influential in shaping the mental health of former caregivers during the post-caregiving phase. The role of co-residence with a care recipient has scarcely been examined; however, it is reasonably predicted to affect former caregivers’ mental health based on previous evidence of its adverse psychological effects.^24^^–^^26^ Previous findings underscore the dynamic and presumably nonlinear nature of the adjustment process in former caregivers’ mental health following caregiving cessation,^18^^,^^19^ advocating the development of a dynamic framework of analysis to better understand these changes.

In this study, we examined the trajectory of former caregivers’ psychological distress (PD), a key measure of mental health, at the cessation of caregiving and in subsequent years using longitudinal data in Japan. Specifically, we investigated the dynamics of PD both before and after the cessation of caregiving. We examined the extent to which these dynamics were influenced by various dimensions of informal caregiving and compared the results of female and male caregivers.

## METHODS

### Study sample

This study used data obtained from a nationwide 17-wave panel survey, “The Longitudinal Survey of Middle-Aged and Older Adults,” conducted by the Japanese Ministry of Health, Labour and Welfare (MHLW) each year from 2005 to 2021. Japan’s Statistics Law requires surveys to be reviewed from statistical, legal, and ethical perspectives. Survey data were obtained from the MHLW with official permission; therefore, ethical approval was not required.

The survey started with a cohort aged 50–59 years (born between 1946 and 1955) in the first wave (2005). In total, 34,240 individuals responded to the survey (response rate: 83.8%). The 2^nd^ to 17^th^ waves of the survey were conducted in early November of each year from 2006 to 2021, and 18,999 respondents remained in the seventeenth wave. No new samples were obtained in this study.

We focused on individuals who started caregiving for at least one of their parents and parents-in-law in the second wave (2006) or later and completed it by the last wave (2021). We excluded those who had already started caregiving in the first wave because we could not determine the duration of caregiving in years. We further concentrated on the period from 1 year before the cessation of caregiving to 3 years after it. Data from the fourth year onwards post-cessation were excluded because PD during those periods was likely influenced by factors other than the cessation of caregiving.^27^ After excluding individuals who missed key variables or dropped out of the survey during the study period, we used 30,538 longitudinal observations of 8,280 individuals (4,439 women and 3,841 men), constituting 25.1% of the initial participants.

### Measures

#### Caregiving-related variables

This study focused on family caregivers who provided informal care to at least one parent or parent-in-law. We examined four dimensions of caregiving: (1) co-residence with a care recipient; (2) duration of caregiving in years; (3) average daily caregiving hours; and (4) engagement in social activities. For the first dimension, we constructed a binary variable for co-residence by allocating one to the respondents who resided with their care-receiving parent in the last year of caregiving and zero to others. Institutionalization (ie, entering a nursing home), which cannot be identified by the survey, was reflected in a change in the binary variable for co-residence from one to zero. In terms of the second dimension, we counted the number of years of caregiving (including the years of suspended caregiving) and constructed a binary variable for extended duration of care by allocating 1 to 3 years or longer and zero to others. For the third dimension, we constructed a binary variable for long-hour care by allocating one to 3 hours or more of care, and zero to others. In the fourth dimension, the survey asked respondents whether they participated in six types of social activities (multiple answers permitted): 1) hobbies or entertainment; 2) sports or physical exercise; 3) community activities; 4) childcare support or educational or cultural activities in the community; 5) support for older adults in the community; and 6) others. We constructed a binary variable for “no social activity” by allocating one to those who did not engage in any type of social activity during the caregiving period. Additionally, we constructed four binary variables for caring for fathers, mothers, fathers-in-law, and mothers-in-law.

#### Psychological distress

We used the Kessler 6-item Psychological Distress Scale (K6) score to measure PD.^28^^,^^29^ The reliability and validity of this tool have been established in Japanese samples.^30^^,^^31^ First, we obtained the respondents’ assessments of PD using the six items from the K6 scale, which include: ‘During the past 30 days, about how often did you feel 1) nervous, 2) hopeless, 3) restless or fidgety, 4) so depressed that nothing could cheer you up, 5) that everything was an effort, and 6) worthless?’ These items are rated on a 5-point scale ranging from 0 (*none of the time*) to 4 (*all of the time*). We then calculated the sum of the reported scores (range: 0–24) and defined this as the K6 score. Higher K6 scores indicate higher levels of PD. Cronbach’s alpha coefficient for this sample was 0.899. We defined PD as a K6 score ≥5, which suggests a mood disorder in a Japanese sample, as validated by previous studies with a sensitivity of 100% and a specificity of 68.7%.^29^^,^^31^ We further considered a higher cut-off point for PD, K6 score ≥13, which suggests severe mental illness.

#### Covariates

We considered sex, age (in years), educational attainment, job status, and bereavement of care-receiving parents as covariates. Regarding educational attainment, we constructed binary variables for graduation from junior high school, high school, junior college, college or higher, and others. We constructed a binary variable for non-paid jobs by allocating one to participants who did not have any paid job during the caregiving period and zero to others. For the bereavement of a care-receiving parent, we constructed a binary variable by allocating one to respondents whose care-receiving parents passed away at the cessation of caregiving and zero to other cases. Furthermore, we included a binary variable for years 2020–21 (16^th^–17^th^ waves) to capture the coronavirus disease 2019 pandemic’ effect.

### Analytic strategy

For the descriptive analysis, we graphically depicted changes in the probability of PD from the pre- to post-caregiving periods separately for female and male caregivers. Regression analyses were performed using a linear mixed model with random intercepts at the individual level. This model explained the trajectory of the probability of PD at the cessation of caregiving and over the subsequent 3 years, initially focusing on the differences between female and male former caregivers:

$$\begin{array}{rl} PD & =\sum_{t=0}^{3}\alpha_{t}t+\sum_{t=0}^{3}\beta_{t}t\times Male+\gamma Male\\ & \;+(\text{care related variables})+(\text{covariates})+e+\varepsilon , \end{array}$$

where *t* indicates the number of years since the cessation of caregiving (with *t* = 0 indicating the year in which caregiving ended), *Male* is a binary variable for a male former caregiver, *e* indicates an individual-level random effect to account for unobserved between-individual heterogeneity and is assumed to be independent and identically distributed across individuals with one common variance, and ε is an error term. We used longitudinal data from each individual, which spanned from 1 year before to 3 years after the cessation of caregiving.

Because all variables (except for age)—both dependent and explanatory—are binary and the regression model is linear, the estimated values of regression coefficients can be interpreted straightforward. Specifically, the estimated value of *α_t_* represents the change in the probability of PD in year *t* (with *t* = 0 at the cessation), from the reference level observed 1 year before the cessation, for female caregivers. The estimated value of *α_t_* + *β_t_* indicates a change in the probability for male caregivers. Accordingly, the estimated value of *α_t_* + *β_t_* + *γ* indicates the male caregivers’ relative position of PD to the female caregivers’ reference level observed 1 year before the cessation of caregiving. The estimated value of *β_t_* + *γ* shows how the probability of PD in male caregivers differed from that in female caregivers.

Considering that a more change in the probability of developing PD after cessation of caregiving was observed among female caregivers (as discussed below), the following analysis focused on female caregivers: We replaced *Male* in the abovementioned model with caregiving-related variables—namely, co-residence, long-hour daily care, extended duration of care, and no social activity—and estimated the model separately for each variable. In the case of co-residence, for instance, the estimated value of *α_t_* indicates the change in the probability of PD after the caregiving cessation for female caregivers who did not reside with a care-receiving parent. The estimated value of *α_t_* + *β_t_* indicates the change for caregivers who lived with the care recipient. The terms *α_t_* + *β_t_* + *γ* and *β_t_* + *γ* can be interpreted in the same way as explained above. To check the robustness of the estimation results, we estimated the same regression models with a higher cut-off point for PD (K6 score ≥13). All statistical analyses were performed using the Stata software package (Release 17; Stata Corp, College Station, TX, USA).

## RESULTS

Table 1 summarizes the key features of caregivers 1 year before caregiving cessation. Compared to male caregivers, female caregivers exhibited a higher probability of PD and had more years of caregiving experience, along with longer daily care hours. However, they were less likely to live with their care-receiving parents. Female caregivers were less likely to have paid jobs but were more likely to engage in social activities. More than half of the caregiving cessations were associated with the bereavement of a care-receiving parent. Regarding PD, its prevalence in our study sample was 36.6%, well above 24.9% in the general Japanese proportion in 2022.^32^

**Table 1.** Key features of caregivers 1 year before the cessation of caregiving

		Female	Male	Total
Age, years	*M*	62.8	63.4	63.1
	*SD*	(4.9)	(4.9)	(4.9)
K6 score (range: 0–24)	*M*	4.5	3.6	4.1
	*SD*	(4.6)	(4.3)	(4.5)

Duration of caregiving, years	*M*	4.5	3.9	4.2
	*SD*	(3.7)	(3.4)	(3.6)
Hours for caregiving per day	*M*	2.7	2.0	2.4
	*SD*	(3.5)	(3.0)	(3.3)

*Probability* (%)				
Psychological distress (K6 score ≥5)		40.8	31.7	36.6
Psychological distress (K6 score ≥13)		6.2	4.4	5.4
Duration of caregiving				
1 year		30.6	37.5	33.8
2 years		11.1	11.1	11.1
≤3 years		58.3	51.5	55.1
Coresided with a care receiver (s)		39.8	50.1	44.6
Caregiving to a father		16.2	19.3	17.7
Caregiving to a mother		49.2	55.6	52.2
Caregiving to a father-in-law		9.7	8.9	9.3
Caregiving to a mother-in-low		31.8	23.4	27.9
Hours for caregiving per day ≥3 hours		22.1	14.7	18.7
Bereavement of a care-receiving parent		57.3	52.5	55.1
No social activity during caregiving		12.4	14.1	13.2
No paid job during caregiving		38.4	18.2	29.0

Educational attainment				
Junior high school		11.5	13.3	12.3
High school		61.6	50.8	56.6
Junior college		16.0	3.0	10.0
College		9.6	31.2	19.6
Other		1.4	1.7	1.5

Number of individuals		4,439	3,841	8,280

K6, Kessler 6-item Psychological Distress Scale.

**Figure 1.**
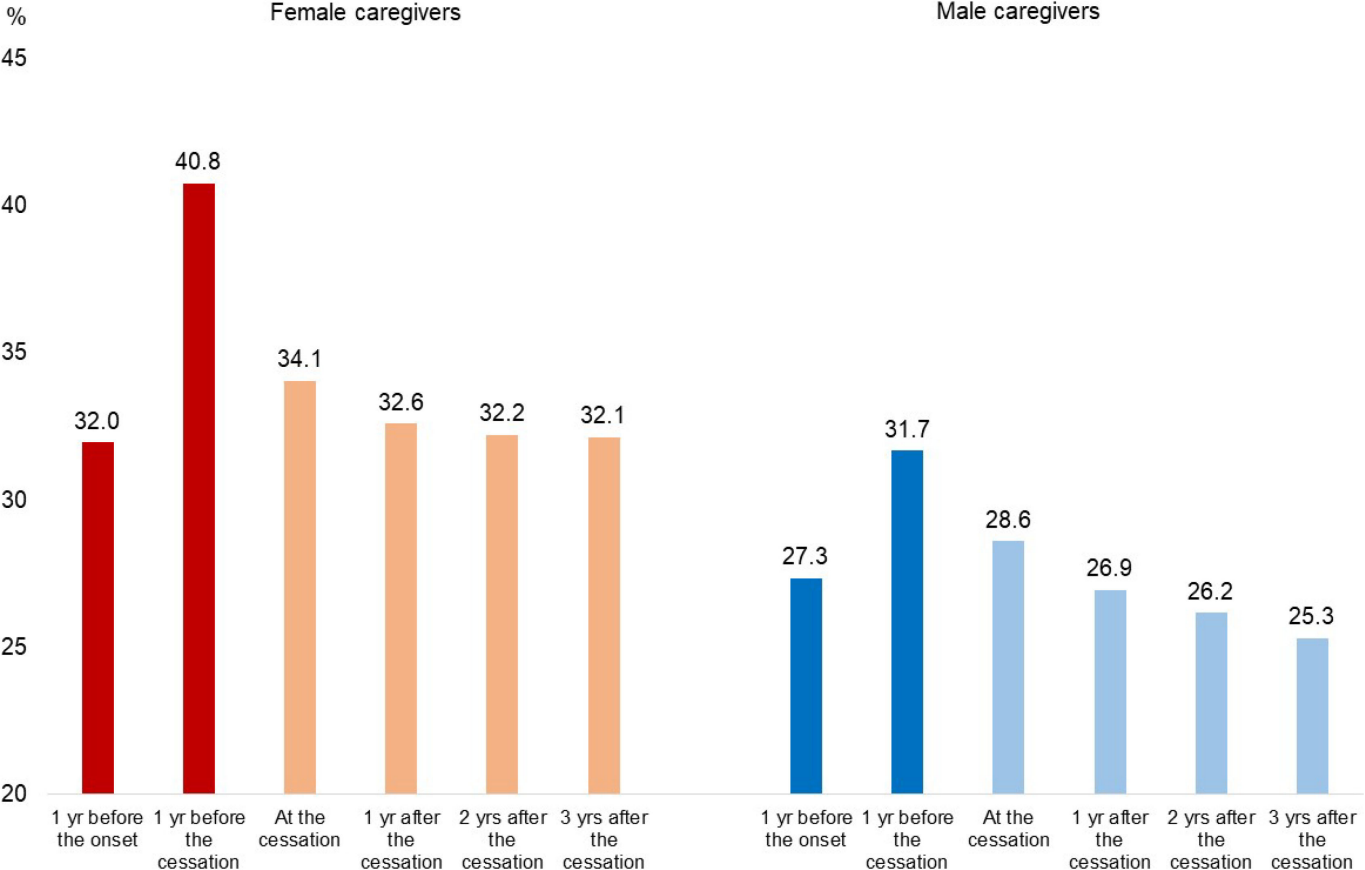
Change in the probability of psychological distress from the pre- to post-caregiving phases. The variables were not adjusted for any other variables.

Figure 1 illustrates the change in the probability of PD from the pre-caregiving phase to the post-caregiving phase without adjusting for other variables. The probability increased substantially during caregiving in both women and men. It decreased sharply upon cessation of caregiving and remained nearly constant over the subsequent 3 years in women, while it kept decreasing after the cessation of caregiving in men. The probability returned nearly to the pre-caregiving level in women and fell below it in men in the third year.

**Figure 2.**
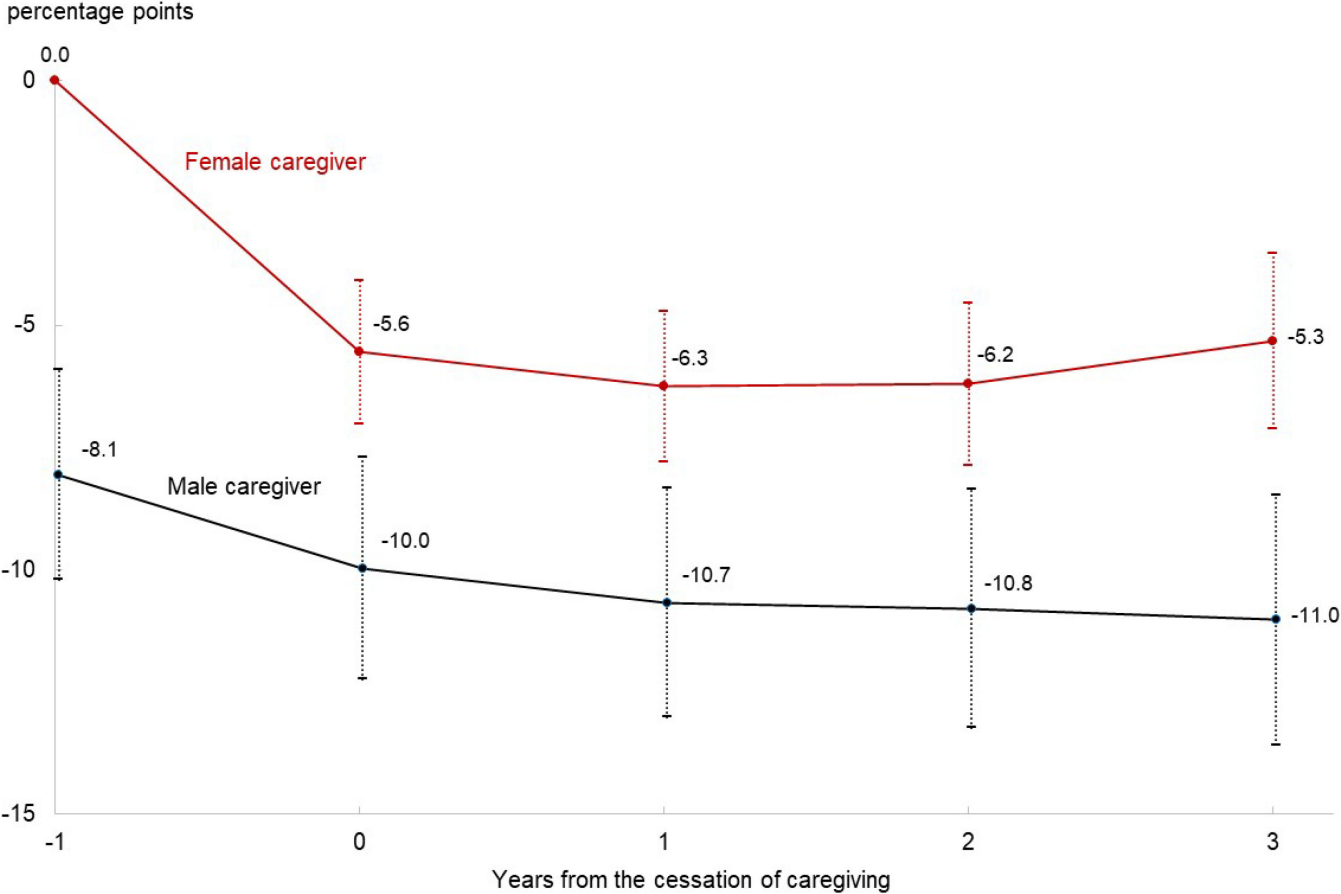
Changes in psychological distress after the cessation of caregiving: female versus male caregivers. Note: The curves indicate the relative positions of the probability of psychological distress to the female caregivers’ level, with 1 year before the cessation of caregiving as a reference point (origin).

Table 2 describes the estimation results obtained from the linear mixed model to explain the probability of PD from 1 year before cessation to 3 years after cessation of caregiving for each individual. The estimated value of *α*_0_ indicates a decrease in the probability of PD by 5.6 percentage points (95% confidence interval [CI], 4.1–7.0%) at the cessation of caregiving for female caregivers, with the probability remaining nearly constant over the subsequent 3 years. A series of estimated values of *β_t_* were consistently and significantly positive, indicating that the pace of reduction in the probability of PD was slower for male than for female caregivers. The reduction in the probability of PD at the cessation of caregiving, calculated by *α*_0_ + *β*_0_, was 1.9 percentage points (95% CI, 0.3–3.5%), which was much smaller than the 5.6 percentage points for women. Moreover, the estimated value of *γ* indicates that the probability of PD 1 year before the cessation of caregiving was 8.1 percentage points lower for male than for female caregivers. The relative position of the male caregivers’ PD, calculated by *α_t_* + *β_t_* + *γ*, remained favorable even after caregiving ended. These results are graphically depicted in Figure 2, which compares the transition of the relative positions of PD probabilities between female and male caregivers.

**Table 2.** Estimation results of the regression model to explain the probability of psychological distress

	Coef.		95% CI
At the cessation of caregiving (*α*_0_)	−0.056	^***^	(−0.070 to −0.041)
1 year after the cessation of caregiving (*α*_1_)	−0.063	^***^	(−0.078 to −0.047)
2 years after the cessation of caregiving (*α*_2_)	−0.062	^***^	(−0.079 to −0.045)
3 years after the cessation of caregiving (*α*_3_)	−0.053	^***^	(−0.071 to −0.035)

At the cessation of caregiving × Male (*β*_0_)	0.036	^***^	(0.015–0.058)
1 year after the cessation of caregiving × Male (*β*_1_)	0.036	^***^	(0.014–0.059)
2 years after the cessation of caregiving × Male (*β*_2_)	0.035	^**^	(0.012–0.058)
3 years after the cessation of caregiving × Male (*β*_3_)	0.024		(0.000–0.048)
Male (*γ*)	−0.081	^***^	(−0.102 to −0.059)

Hours for caregiving per day ≥3 hours	0.047	^***^	(0.028–0.066)
Duration of caregiving ≥3 years	0.055	^***^	(0.037–0.074)

Co-resided with a care-receiver	0.011		(−0.006 to 0.028)
Caregiving to father	0.039	^*^	(0.004–0.074)
Caregiving to mother	0.038	^*^	(0.004–0.073)
Caregiving to father-in-law	0.042	^*^	(0.004–0.080)
Caregiving to mother-in-low	0.011		(−0.024 to 0.046)
Bereavement of a care-receiving parent	−0.007		(−0.024 to 0.010)
No social activity during caregiving	0.083	^***^	(0.055–0.110)
No paid job during caregiving	0.023	^*^	(0.004–0.042)
Age (years)	−0.005	^***^	(−0.008 to −0.003)
Educational attainment (ref. = college or above)			
Junior high school	0.049	^**^	(0.018–0.079)
High school	0.034	^**^	(0.011–0.057)
Junior college	−0.014		(−0.048 to 0.020)
Other	0.075	^*^	(0.006–0.143)
COVID-19 pandemic	0.018	^*^	(0.003–0.032)

Number of individuals	8,513		
Number of observations	30,538		

Post-regression calculation			
*α*_0_ + *β*_0_	−0.019	^*^	(−0.035 to −0.003)
*α*_1_ + *β*_1_	−0.026	^**^	(−0.043 to −0.009)
*α*_2_ + *β*_2_	−0.027	^**^	(−0.045 to −0.010)
*α*_3_ + *β*_3_	−0.030	^**^	(−0.049 to −0.011)
*α*_0_ + *β*_0_ + *γ*	−0.100	^***^	(−0.122 to −0.077)
*α*_1_ + *β*_1_ + *γ*	−0.107	^***^	(−0.130 to −0.083)
*α*_2_ + *β*_2_ + *γ*	−0.108	^***^	(−0.132 to −0.084)
*α*_3_ + *β*_3_ + *γ*	−0.110	^***^	(−0.136 to −0.085)

CI, confidence interval; COVID-19, coronavirus disease 2019.

^***^P < 0.001, ^**^P < 0.01, ^*^P < 0.05.

In addition to the main results, Table 2 shows that long-hour care, long duration, and no social activity increased the probability of PD in caregivers, whereas the bereavement of the care-receiving parent did not exhibit a significant impact. Co-residence did not have any impact, although it increased PD when the sample was limited to female caregivers.

**Figure 3.**
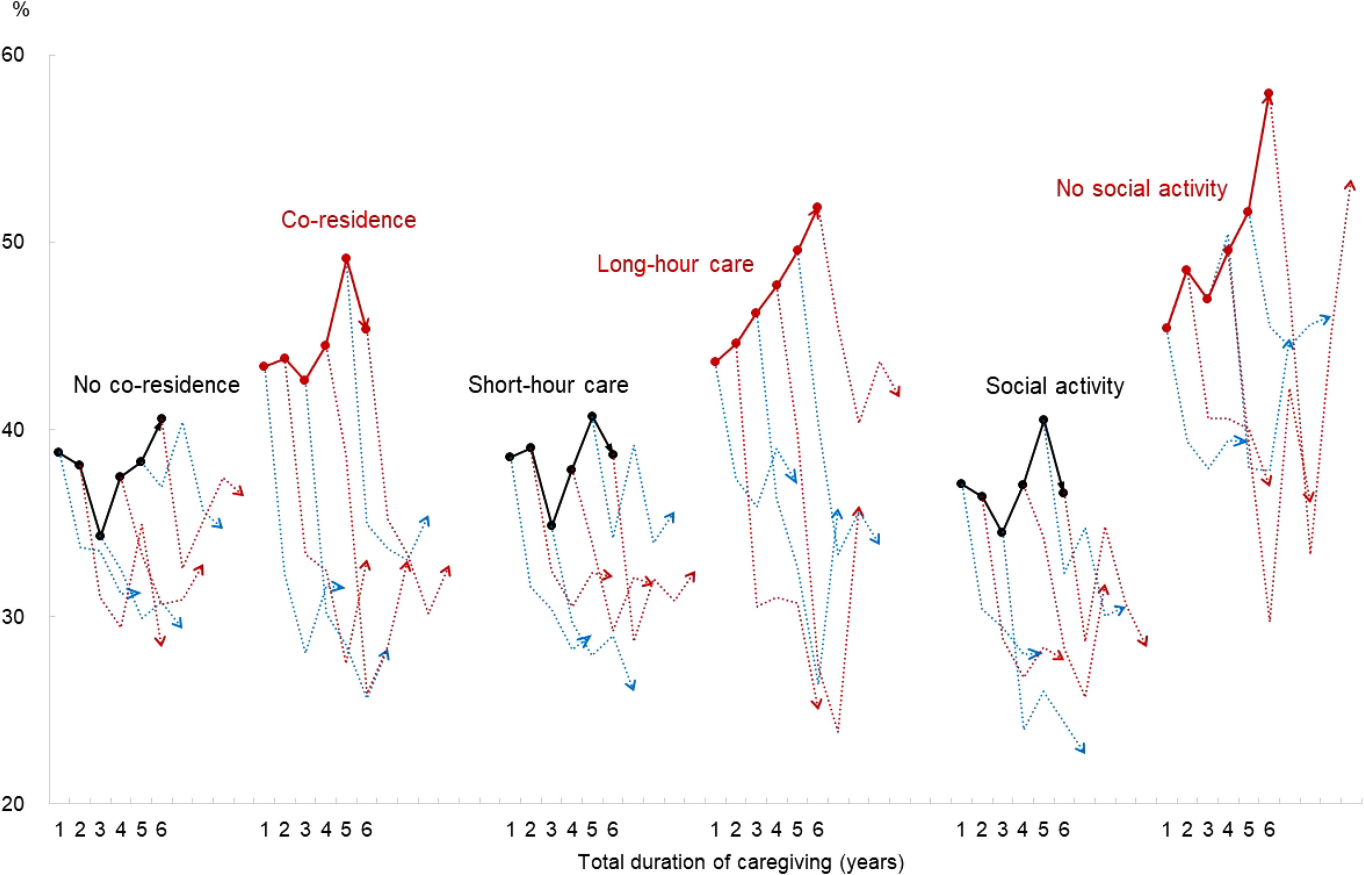
Transition of the probability of psychological distress after the cessation of caregiving by type of caregiving for female caregivers

Figure 3 shows how the transition of PD in the post-caregiving phase was associated with various caregiving factors without controlling for other factors. The solid lines indicate changes in the probability of PD 1 year before caregiving ended across different caregiving durations. The dotted lines show how the probability of PD evolved from the time of cessation of caregiving through the subsequent 3 years, corresponding to different caregiving durations.

The four findings discussed in this figure are noteworthy. First, longer caregiving duration was associated with an increased probability of PD, especially in cases involving co-residence, long hours of care, and no social activity. Conversely, this trend was less pronounced in situations with no co-residence, short hours of care, or social activity. Second, the cessation of caregiving triggered a sharp drop in PD across all scenarios, with a decrease observed involving co-residence, long-hour care, and no social activity, which are factors associated with a higher probability of PD before caregiving ends. Third, the discrepancy in the probability of PD between those with and without co-residence narrowed significantly after caregiving ceased, whereas the disparity remained substantial between those who engaged in social activities and those who did not. The results of care intensity were mixed, falling between the patterns observed in co-residence and social activity.

**Figure 4.**
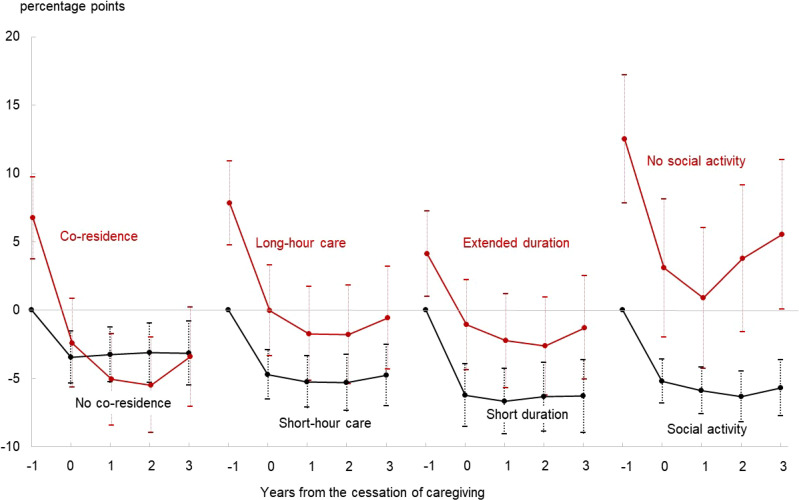
Transition of psychological distress after the cessation of caregiving by type of caregiving for female caregivers

Figure 4 depicts how the transition of the probability of PD was associated with co-residence, hours of care, duration in years, and engagement in social activity for women, based on the estimated values of *α_t_*, *β_t_*, and *γ* in the mixed models (the full results are represented in eTable 1). The following three main findings were obtained: First, consistent with the results shown in Figure 2 and Figure 3, the probability of PD decreased sharply at the cessation of caregiving and remained almost constant in subsequent years across scenarios for women. Second, the relatively unfavorable impact of co-residence disappeared quickly at the cessation of caregiving, while the impacts of no social activity, extended duration, and, to a lesser extent, long-hour care persisted after caregiving ceased. Third, a few reversals in probability were observed in scenarios involving co-residence, long-hour care, extended duration, and no social activity following a few initial improvements, whereas scenarios involving no co-residence, short-hour care, short-year duration, or social activity exhibited almost flat trends.

We repeated the same analysis for male caregivers and reported the results in eTable 2 and eFigure 1. As seen in these table and figure, the reduction in the probability of PD at caregiving cessation was generally more limited for male caregivers than for female caregivers. Consequently, the relatively unfavorable positions of caregivers with these features did not substantially change even after the cessation of caregiving. Moreover, there was no significant difference in the progression of PD between residents and non-residents.

Finally, we conducted the sensitivity analysis with a higher cut-off point for PD (K6 score ≥13). The key results are presented in eFigure 2, eFigure 3, eFigure 4, eFigure 5, eTable 3, eTable 4, and eTable 5. As seen in these tables and figures, the transitions of PD after the cessation of caregiving presented patterns largely similar to those for K6 score ≥5, but they were more mixed and less affected by caregiving conditions except for engagement in social activity.

## DISCUSSION

This study examined the trajectory of the likelihood of PD up to 3 years following the cessation of caregiving among individuals who had been caring for older parents. In line with many previous studies showing the adverse impact of informal caregiving on family caregivers’ mental health, we confirmed that family caregivers faced much heavier PD compared to the general Japanese population. More remarkably, we observed that the cessation of caregiving triggered a sharp reduction in the probability of PD among caregivers. Relief from caregiving was observed even after controlling for the bereavement of care-receiving parents. Hence, our observations support the view that exiting caregiving is favorable to former caregivers’ mental health.^8^^,^^11^^–^^14^ Relief from caregiver burden was more substantial for female caregivers than for male caregivers. This result aligns with women bearing heavier burdens for caregiving in terms of intensity and duration, which is consistent with the observation that a much larger proportion of female caregivers had no paid jobs than male caregivers. The observed trajectory of PD was nonlinear,^17^ with a sharp decrease at cessation, followed by a gradual improvement in subsequent years.

Although caregivers’ mental health tended to improve smoothly after the cessation of caregiving, the transition of PD depended heavily on the dimensions of caregiving. For female caregivers, the adverse impact of co-residence disappeared quickly in response to the cessation of caregiving. In contrast, the unfavorable impacts of no social activity, long duration, and long hours of care tended to persist for years after caregiving ended. Higher levels of PD associated with no social activity and a long duration of care were not fully relieved by the cessation of caregiving. Long-lasting effects of no social activity, long duration, and long-hour care were observed, albeit less clearly, for male caregivers.

The finding that former caregivers’ mental health was closely associated with caregiving conditions corroborates observations from studies outside Japan.^22^^,^^23^ When individuals provide care over several years, they often sacrifice other aspects of their lives, particularly their social connections, which can be difficult to regain.^33^ The objective caregiving burdens, measured by care demands and caregiving workload, make family caregivers socially isolated, which in turn increases their risk of experiencing depressive symptoms.^34^ Future policymaking and social work practice should target these vulnerable groups by providing support for informal caregivers, such as establishing respite care programs and creating support networks in the community.

This study had several limitations. First, we did not fully control for potential bias due to endogeneity. Although we treated cessation of caregiving as an exogenous variable, which may be justifiable, especially if accompanied by the bereavement of a care-receiving parent, the possibility that the deterioration of caregivers’ mental health could lead to the cessation of caregiving cannot be ruled out. Second, we did not adjust for the attrition bias. Extremely high levels of PD may have caused participants to drop out of the survey. If this were the case, the observed reduction in PD at the time of caregiving cessation would have been underestimated. Third, we did not directly examine the severity of care needed because of a lack of data availability, although we considered the hours of daily care and the duration of caregiving in years. Fourth, we did not analyze changes in former caregivers’ behaviors. The cessation of caregiving may encourage individuals to engage in social activities,^14^^,^^35^ which may affect their mental health. Finally, as suggested by more nuanced results of the sensitivity analysis with a higher cut-off point for PD, this study did not address pathological aspects of caregivers’ mental health.

Despite these limitations, the results underscore the significant psychological burden of informal caregiving on family caregivers. This was evidenced by a reduction in the likelihood of PD following the cessation of caregiving. However, it is equally important to note that the unfavorable impacts associated with a lack of social activity, long duration, and extensive hours of caregiving tended to persist even after caregiving ended. These findings suggest that changes in mental health after caregiving cessation should be considered when developing policy measures to support informal caregivers. Excessive reliance on family caregiving should be avoided in public long-term care schemes and policies aimed at preventing caregivers’ social isolation must be implemented.

## SUPPLEMENTARY MATERIAL

eTable 1. Estimated impact of the cessation of caregiving on female caregivers’ psychological distress

eTable 2. Estimated impact of the end of caregiving on male caregivers’ psychological distress

eTable 3. Estimation results of the regression model to explain the probability of psychological distress (K6 ≥13)

eTable 4. Estimated impact of the cessation of caregiving on female caregivers’ psychological distress (K6 ≥13)

eTable 5. Estimated impact of the cessation of caregiving on male caregivers’ psychological distress (K6 ≥13)

eFigure 1. Evolution of PD after the cessation of caregiving by type of caregiving for male caregivers

eFigure 2. Change in the probability of psychological distress (K6 ≥13) from the pre- to post-caregiving phases

eFigure 3. Changes in psychological distress (K6 ≥13) after the cessation of caregiving: female vs male caregivers:

eFigure 4. Transition of psychological distress (K6 ≥13) after the cessation of caregiving by type of caregiving for female caregivers

eFigure 5. Transition of psychological distress (K6 ≥13) after the cessation of caregiving by type of caregiving for male caregivers
