# Supplementary material for: Can Cessation of Caregiving for Parents Relieve Family Caregivers’ Psychological Distress? A Longitudinal Study Using 17-wave Nationwide Survey Data in Japan
Source: J Epidemiol. 2025 Apr 5;35(4):187–94. doi: 10.2188/jea.JE20240190 (PMC11882349; doi:10.2188/jea.JE20240190)

eTable 1. Estimated impact of the cessation of caregiving on female caregivers' psychological distress

	Co-residence		Long-hour care		Extended duration		No social activity	
	Coef.	95% CI	*Coef.*	95% CI	*Coef.*	95% CI	*Coef.*	95% CI
α_0	–0.034***	(–0.053 to –0.015)	–0.047***	(–0.065 to –0.029)	–0.062***	(–0.085 to –0.039)	–0.052***	(–0.068 to –0.036)
α_1	–0.033***	(–0.053 to –0.012)	–0.052***	(–0.071 to –0.033)	–0.066***	(–0.090 to –0.043)	–0.059***	(–0.076 to –0.042)
α_2	–0.031**	(–0.053 to –0.009)	–0.053***	(–0.074 to –0.033)	–0.063***	(–0.089 to –0.038)	–0.063***	(–0.082 to –0.044)
α_3	–0.031**	(–0.055 to –0.008)	–0.048***	(–0.070 to –0.025)	–0.063***	(–0.090 to –0.036)	–0.057***	(–0.077 to –0.037)
β_0	–0.057***	(–0.088 to –0.026)	–0.031†	(–0.064 to 0.001)	0.010	(–0.020 to 0.040)	–0.042	(–0.092 to 0.007)
β_1	–0.086***	(–0.117 to –0.054)	–0.043*	(–0.077 to –0.009)	0.003	(–0.028 to 0.034)	–0.057*	(–0.108 to –0.007)
β_2	–0.091***	(–0.124 to –0.058)	–0.043*	(–0.078 to –0.007)	–0.004	(–0.037 to 0.028)	–0.024	(–0.077 to 0.028)
β_3	–0.070***	(–0.105 to –0.036)	–0.036†	(–0.073 to 0.001)	0.009	(–0.025 to 0.043)	–0.013	(–0.067 to 0.041)
γ	0.067***	(0.037–0.097)	0.078***	(0.047–0.109)	0.041**	(0.010–0.072)	0.125***	(0.078–0.172)
$\beta_0 + \gamma$	0.010	(–0.022 to 0.043)	0.047**	(0.013–0.081)	0.051**	(0.018–0.085)	0.083**	(0.032–0.134)
$\beta_1 + \gamma$	–0.018	(–0.052 to 0.016)	0.035*	(0.000–0.070)	0.044*	(0.010–0.078)	0.068*	(0.015–0.120)
$\beta_2 + \gamma$	–0.024	(–0.059 to 0.011)	0.035†	(–0.001 to 0.072)	0.037*	(0.002–0.072)	0.101***	(0.046–0.155)
$\beta_3 + \gamma$	–0.003	(–0.039 to 0.034)	0.042*	(0.004–0.080)	0.050**	(0.014–0.086)	0.112***	(0.057–0.167)
$\alpha_0 + \beta_0 + \gamma$	–0.024	(–0.056 to 0.008)	0.000	(–0.034 to 0.033)	–0.011	(–0.043 to 0.022)	0.031	(–0.020 to 0.081)
$\alpha_1 + \beta_1 + \gamma$	–0.051**	(–0.084 to –0.017)	–0.017	(–0.052 to 0.017)	–0.022	(–0.057 to 0.012)	0.009	(–0.043 to 0.060)
$\alpha_2 + \beta_2 + \gamma$	–0.055**	(–0.090 to –0.020)	–0.018	(–0.054 to 0.018)	–0.026	(–0.062 to 0.010)	0.038	(–0.016 to 0.091)
$\alpha_3 + \beta_3 + \gamma$	–0.034†	(–0.071 to 0.002)	–0.006	(–0.043 to 0.032)	–0.013	(–0.051 to 0.025)	0.055*	(0.001–0.110)

CI, confidence interval.

*** P<0.001, ** P<0.01, * P<0.05, † P<0.1

eTable 2. Estimated impact of the end of caregiving on male caregivers' psychological distress

	Co-residence		Long-hour care		Extended duration		No social activity	
	Coef.	95% CI	*Coef.*	95% CI	*Coef.*	95% CI	*Coef.*	95% CI
α_0	–0.009	(–0.031 to 0.012)	–0.021 *	(–0.039 to –0.004)	–0.025 *	(–0.047 to –0.003)	–0.015	(–0.032 to 0.001)
α_1	–0.017	(–0.039 to 0.006)	–0.031 ***	(–0.050 to –0.013)	–0.015	(–0.038 to 0.007)	–0.019 *	(–0.036 to –0.001)
α_2	–0.011	(–0.035 to 0.014)	–0.028 **	(–0.048 to –0.008)	–0.013	(–0.037 to 0.011)	–0.020 *	(–0.039 to –0.001)
α_3	–0.004	(–0.030 to 0.022)	–0.031 **	(–0.054 to –0.009)	–0.018	(–0.044 to 0.007)	–0.019	(–0.040 to 0.002)
β_0	–0.016	(–0.047 to 0.014)	0.017	(–0.020 to 0.053)	0.016	(–0.015 to 0.046)	–0.018	(–0.067 to 0.031)
β_1	–0.010	(–0.042 to 0.022)	0.043 *	(0.005–0.080)	–0.013	(–0.045 to 0.018)	–0.029	(–0.079 to 0.022)
β_2	–0.021	(–0.054 to 0.012)	0.032	(–0.008 to 0.071)	–0.017	(–0.050 to 0.016)	–0.010	(–0.063 to 0.044)
β_3	–0.033	(–0.067 to 0.001)	0.049 *	(0.008–0.090)	–0.004	(–0.039 to 0.030)	–0.017	(–0.071 to 0.037)
γ	0.013	(–0.016 to 0.042)	0.013	(–0.021 to 0.047)	0.072 ***	(0.042–0.103)	0.074 ***	(0.028–0.120)
$\beta_0 + \gamma$	–0.003	(–0.035 to 0.029)	0.030	(–0.007 to 0.067)	0.088 ***	(0.055–0.121)	0.056 *	(0.006–0.106)
$\beta_1 + \gamma$	0.003	(–0.029 to 0.036)	0.056 **	(0.018–0.094)	0.059 ***	(0.025–0.093)	0.046	(–0.006 to 0.097)
$\beta_2 + \gamma$	–0.007	(–0.042 to 0.027)	0.045 *	(0.005–0.085)	0.055 **	(0.020–0.091)	0.065 *	(0.010–0.119)
$\beta_3 + \gamma$	–0.020	(–0.055 to 0.016)	0.062 **	(0.021–0.104)	0.068 ***	(0.032–0.105)	0.058 *	(0.002–0.113)
$\alpha_0 + \beta_0 + \gamma$	–0.012	(–0.043 to 0.019)	0.009	(–0.028 to 0.045)	0.063 ***	(0.030–0.096)	0.041	(–0.009 to 0.091)
$\alpha_1 + \beta_1 + \gamma$	–0.014	(–0.045 to 0.018)	0.024	(–0.013 to 0.062)	0.044 *	(0.009–0.079)	0.027	(–0.024 to 0.078)
$\alpha_2 + \beta_2 + \gamma$	–0.018	(–0.051 to 0.015)	0.017	(–0.022 to 0.056)	0.043 *	(0.006–0.079)	0.045	(–0.009 to 0.099)
$\alpha_3 + \beta_3 + \gamma$	–0.024	(–0.059 to 0.011)	0.031	(–0.010 to 0.072)	0.050 *	(0.011–0.089)	0.039	(–0.016 to 0.094)

CI, confidence interval.
*** $P<0.001$, ** $P<0.01$, * $P<0.05$

eTable 3. Estimation results of the regression model to explain the probability of psychological distress (K6 ≥13)

	Coef.			95% CI	
At the cessation of caregiving (α_0)	0.013	***	(–0.020	to	–0.005)
1 year after the cessation of caregiving (α_1)	–0.007		(–0.015	to	0.000)
2 years after the cessation of caregiving (α_2)	–0.011	**	(–0.019	to	–0.003)
3 years after the cessation of caregiving (α_3)	–0.009	*	(–0.018	to	0.000)
At the cessation of caregiving × Male (β_0)	0.013	*	(0.002	–	0.023)
1 year after the cessation of caregiving × Male (β_1)	0.005		(–0.006	to	0.016)
2 years after the cessation of caregiving × Male (β_2)	0.007		(–0.004	to	0.019)
3 years after the cessation of caregiving × Male (β_3)	0.009		(–0.003	to	0.020)
Male (γ)	–0.012	*	(–0.021	to	–0.002)
Hours for caregiving per day ≥3 hours	0.016	***	(0.008	–	0.024)
Duration of caregiving ≥3 years	0.011	**	(0.003	–	0.019)
Co–resided with a care–receiver	0.004		(–0.003	to	0.011)
Caregiving to father	0.021	**	(0.006	–	0.036)
Caregiving to mother	0.028	***	(0.014	–	0.043)
Caregiving to father–in–law	0.020	*	(0.004	–	0.036)
Caregiving to mother–in–low	0.021	**	(0.006	–	0.036)
Bereavement of a care–receiving parent	0.001		(–0.006	to	0.008)
No social activity during caregiving	0.038	***	(0.026	–	0.049)
No paid job during caregiving	0.014	***	(0.006	–	0.022)
Age, years	–0.002	***	(–0.003	to	–0.001)
Educational attainment (ref. = college or above)					
Junior high school	0.014	*	(0.001	–	0.026)
High school	0.016	***	(0.006	–	0.025)
Junior college	0.003		(–0.012	to	0.017)
Other	0.005		(–0.024	to	0.034)
COVID-19 pandemic	0.004		(–0.003	to	0.011)
Number of individuals	8,513				
Number of observations	30,538				
Post-regression calculation					
$\alpha_0 + \beta_0$	0.000		(–0.008	to	0.008)
$\alpha_1 + \beta_1$	–0.002		(–0.011	to	0.006)
$\alpha_2 + \beta_2$	–0.004		(–0.012	to	0.005)
$\alpha_3 + \beta_3$	0.000		(–0.010	to	0.009)
$\alpha_0 + \beta_0 + \gamma$	–0.011	*	(–0.022	to	–0.001)
$\alpha_1 + \beta_1 + \gamma$	–0.014	**	(–0.024	to	–0.004)
$\alpha_2 + \beta_2 + \gamma$	–0.015	**	(–0.026	to	–0.004)
$\alpha_3 + \beta_3 + \gamma$	–0.012	*	(–0.023	to	–0.001)

CI, confidence interval; COVID-19, coronavirus disease 2019.

*** P<0.001, ** P<0.01, * P<0.05

eTable 4. Estimated impact of the cessation of caregiving on female caregivers' psychological distress (K6 ≥13)

	Coresidence		Long-hour care		Extended duration		No social activity	
	Coef.	95% CI	*Coef.*	95% CI	*Coef.*	95% CI	*Coef.*	95% CI
α_0	–0.004	(–0.013 to 0.006)	–0.008	(–0.017 to 0.001)	–0.013 *	(–0.025 to –0.001)	–0.012 **	(–0.020 to –0.003)
α_1	–0.001	(–0.011 to 0.009)	–0.001	(–0.011 to 0.008)	–0.010	(–0.022 to 0.002)	–0.006	(–0.015 to 0.002)
α_2	0.002	(–0.009 to 0.013)	0.001	(–0.009 to 0.011)	–0.015 *	(–0.028 to –0.003)	–0.008	(–0.017 to 0.001)
α_3	–0.001	(–0.012 to 0.011)	–0.001	(–0.012 to 0.010)	–0.012	(–0.025 to 0.002)	–0.010	(–0.019 to 0.000)
β_0	–0.024 **	(–0.040 to –0.008)	–0.015	(–0.032 to 0.002)	0.000	(–0.015 to 0.016)	–0.013	(–0.038 to 0.012)
β_1	–0.017 *	(–0.033 to 0.000)	–0.023 **	(–0.040 to –0.006)	0.004	(–0.012 to 0.020)	–0.016	(–0.042 to 0.010)
β_2	–0.036 ***	(–0.053 to –0.019)	–0.044 ***	(–0.062 to –0.025)	0.007	(–0.010 to 0.023)	–0.037 **	(–0.064 to –0.010)
β_3	–0.024 **	(–0.041 to –0.006)	–0.032 ***	(–0.051 to –0.013)	0.003	(–0.014 to 0.021)	–0.004	(–0.032 to 0.023)
γ	0.020 **	(0.007– 0.034)	0.004	(–0.007 to 0.015)	0.010	(–0.005 to 0.024)	0.064 ***	(0.042– 0.086)
$\beta_0 + \gamma$	–0.004	(–0.019 to 0.011)	0.023 **	(0.007– 0.038)	0.010	(–0.005 to 0.025)	0.051 ***	(0.027– 0.075)
$\beta_1 + \gamma$	0.004	(–0.012 to 0.019)	0.015	(–0.001 to 0.031)	0.014	(–0.002 to 0.029)	0.048 ***	(0.024– 0.072)
$\beta_2 + \gamma$	–0.015	(–0.032 to 0.001)	–0.006	(–0.023 to 0.011)	0.016 *	(0.000– 0.033)	0.027 *	(0.002– 0.053)
$\beta_3 + \gamma$	–0.003	(–0.021 to 0.014)	0.006	(–0.012 to 0.024)	0.013	(–0.004 to 0.030)	0.060 ***	(0.034– 0.086)
$\alpha_0 + \beta_0 + \gamma$	–0.007	(–0.022 to 0.007)	0.014	(–0.001 to 0.030)	–0.003	(–0.018 to 0.012)	0.040 ***	(0.016 to 0.063)
$\alpha_1 + \beta_1 + \gamma$	0.002	(–0.013 to 0.018)	0.014	(–0.002 to 0.030)	0.004	(–0.012 to 0.019)	0.042 ***	(0.018 to 0.066)
$\alpha_2 + \beta_2 + \gamma$	–0.013	(–0.030 to 0.003)	–0.005	(–0.021 to 0.012)	0.001	(–0.015 to 0.017)	0.019	(–0.006 to 0.045)
$\alpha_3 + \beta_3 + \gamma$	–0.004	(–0.021 to 0.013)	0.005	(–0.012 to 0.023)	0.001	(–0.016 to 0.018)	0.050 ***	(0.024– 0.076)

CI, confidence interval.
*** $P<0.001$, ** $P<0.01$, * $P<0.05$

eTable 5. Estimated impact of the cessation of caregiving on male caregivers' psychological distress (K6 ≥13)

	Coresidence		Long-hour care		Extended duration		No social activity	
	Coef.	95% CI	Coef.	95% CI	Coef.	95% CI	Coef.	95% CI
α_0	0.000	(–0.010 to 0.010)	–0.003	(–0.011 to 0.005)	–0.006	(–0.016 to 0.004)	0.001	(–0.007 to 0.009)
α_1	–0.002	(–0.013 to 0.008)	–0.002	(–0.011 to 0.007)	0.000	(–0.010 to 0.011)	–0.003	(–0.011 to 0.005)
α_2	0.000	(–0.012 to 0.011)	–0.003	(–0.012 to 0.006)	–0.006	(–0.017 to 0.005)	–0.003	(–0.012 to 0.006)
α_3	0.001	(–0.011 to 0.013)	–0.001	(–0.011 to 0.009)	–0.001	(–0.013 to 0.010)	0.002	(–0.007 to 0.011)
β_0	0.002	(–0.013 to 0.016)	0.016	(–0.001 to 0.033)	0.014	(–0.001 to 0.028)	–0.005	(–0.028 to 0.018)
β_1	0.001	(–0.013 to 0.016)	0.002	(–0.015 to 0.020)	–0.004	(–0.019 to 0.011)	0.012	(–0.012 to 0.035)
β_2	–0.004	(–0.020 to 0.011)	0.003	(–0.016 to 0.021)	0.007	(–0.008 to 0.023)	0.005	(–0.020 to 0.030)
β_3	0.000	(–0.016 to 0.016)	0.010	(–0.009 to 0.029)	0.006	(–0.010 to 0.022)	–0.005	(–0.030 to 0.021)
γ	0.004	(–0.008 to 0.017)	0.004	(–0.010 to 0.019)	0.008	(–0.006 to 0.021)	0.021 *	(0.002– 0.041)
$\beta_0 + \gamma$	0.006	(–0.008 to 0.020)	0.004	(–0.010 to 0.019)	0.021 **	(0.007– 0.036)	0.016	(–0.006 to 0.038)
$\beta_1 + \gamma$	0.006	(–0.008 to 0.020)	0.017 *	(0.002– 0.033)	0.004	(–0.011 to 0.018)	0.033 **	(0.011– 0.055)
$\beta_2 + \gamma$	0.000	(–0.015 to 0.015)	0.021 *	(0.004– 0.037)	0.015	(–0.001 to 0.030)	0.026 *	(0.002– 0.050)
$\beta_3 + \gamma$	0.005	(–0.011 to 0.020)	0.005	(–0.012 to 0.021)	0.013	(–0.003 to 0.029)	0.017	(–0.008 to 0.041)
$\alpha_0 + \beta_0 + \gamma$	0.006	(–0.008 to 0.019)	0.004	(–0.013 to 0.021)	0.015 *	(0.001– 0.029)	0.017	(–0.005 to 0.039)
$\alpha_1 + \beta_1 + \gamma$	0.004	(–0.010 to 0.017)	0.007	(–0.010 to 0.025)	0.004	(–0.011 to 0.019)	0.030 **	(0.008– 0.052)
$\alpha_2 + \beta_2 + \gamma$	0.000	(–0.014 to 0.014)	0.013	(–0.005 to 0.031)	0.009	(–0.007 to 0.025)	0.023	(0.000– 0.047)
$\alpha_3 + \beta_3 + \gamma$	0.006	(–0.009 to 0.021)	0.014	(–0.004 to 0.033)	0.012	(–0.005 to 0.028)	0.019	(–0.005 to 0.043)

CI, confidence interval.
*** P<0.001, ** P<0.01, * P<0.05

eFigure 1. Evolution of PD after the cessation of caregiving by type of caregiving for male caregivers

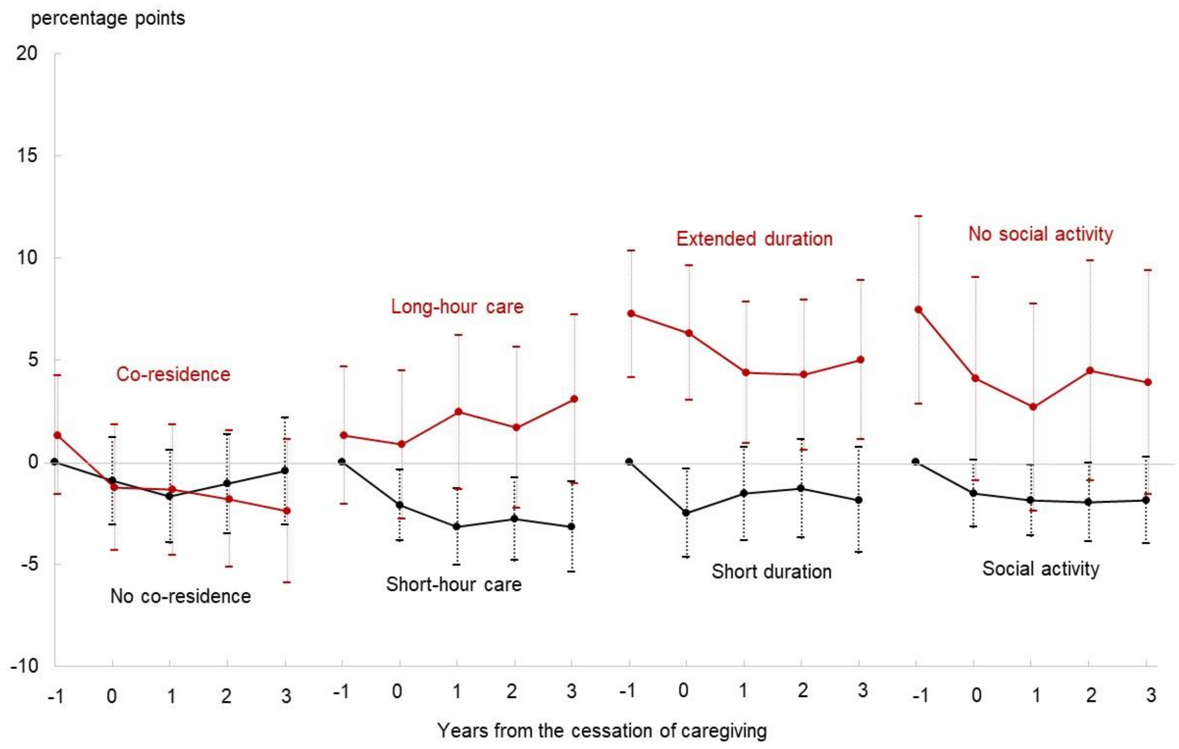

eFigure 2. Change in the probability of psychological distress (K6 ≥13) from the pre- to post-caregiving phases

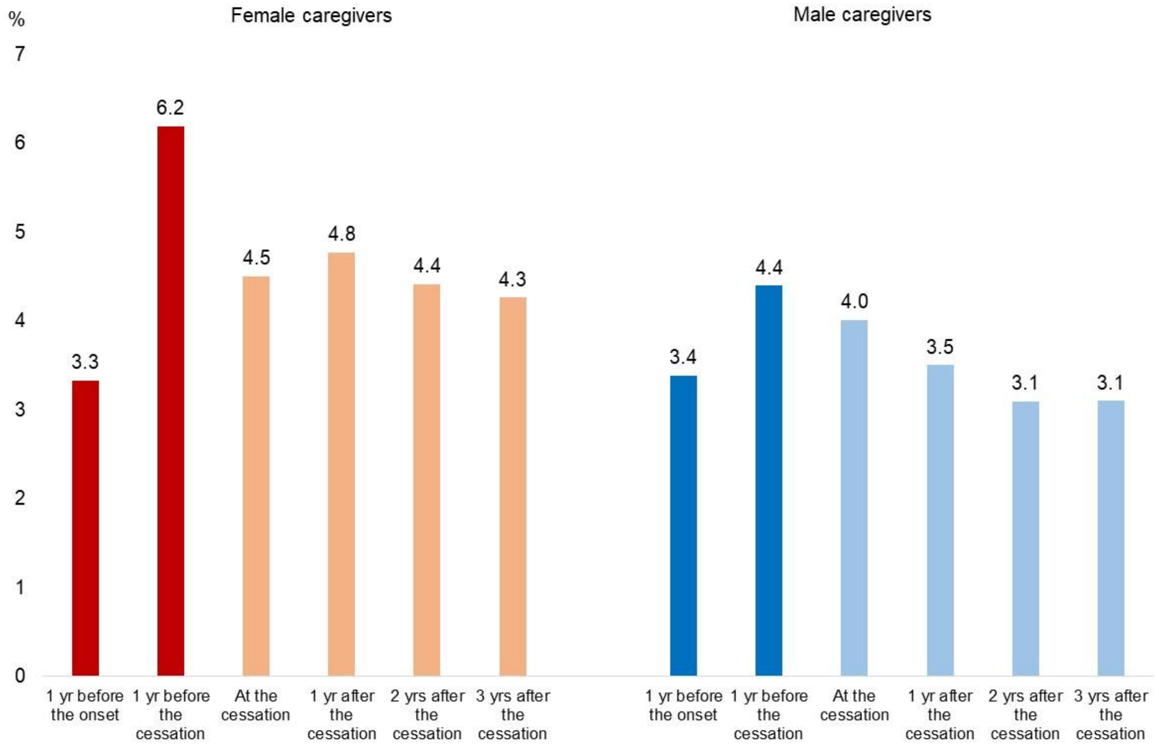

eFigure 3. Changes in psychological distress (K6 ≥13) after the cessation of caregiving: female vs. male caregivers:

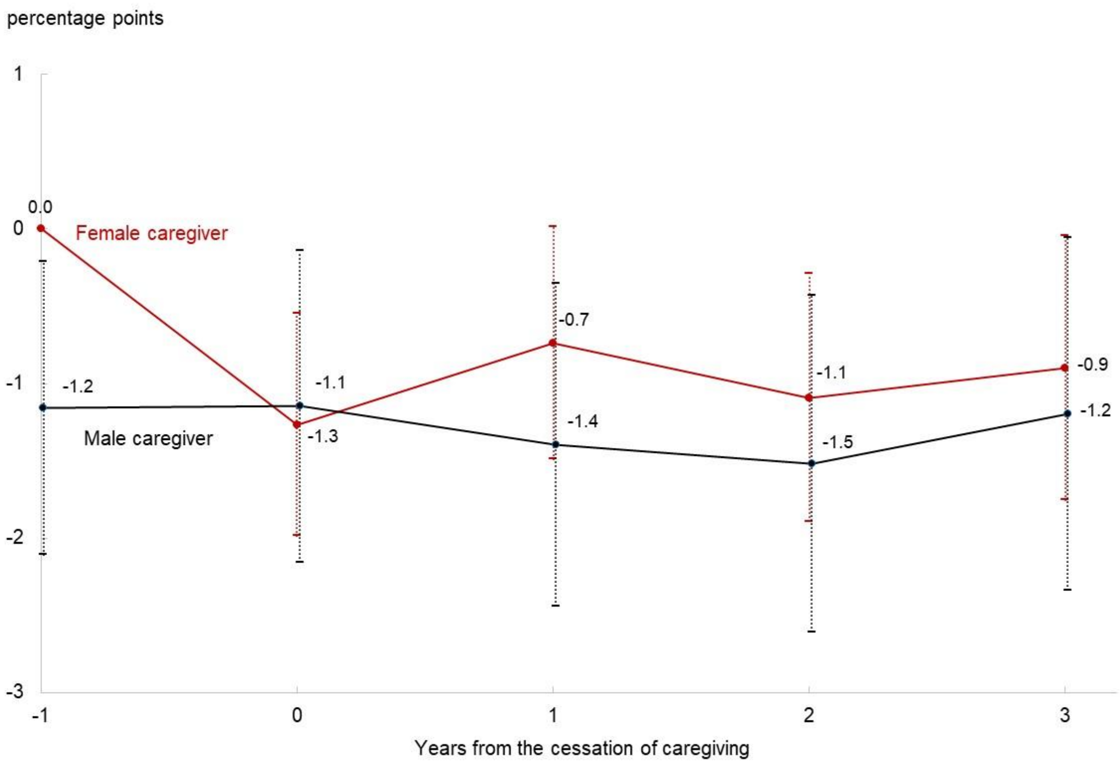

eFigure 4. Transition of psychological distress (K6 ≥13) after the cessation of caregiving by type of caregiving for female caregivers

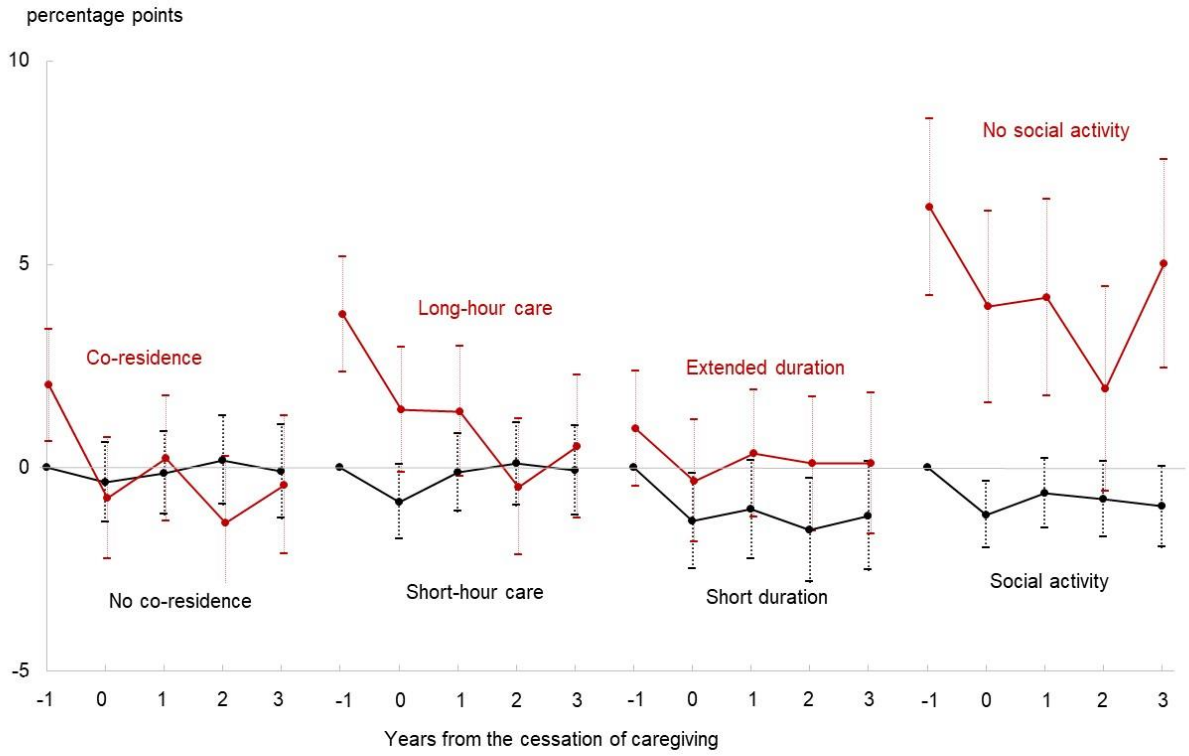

eFigure 5. Transition of psychological distress (K6 ≥13) after the cessation of caregiving by type of caregiving for male caregivers

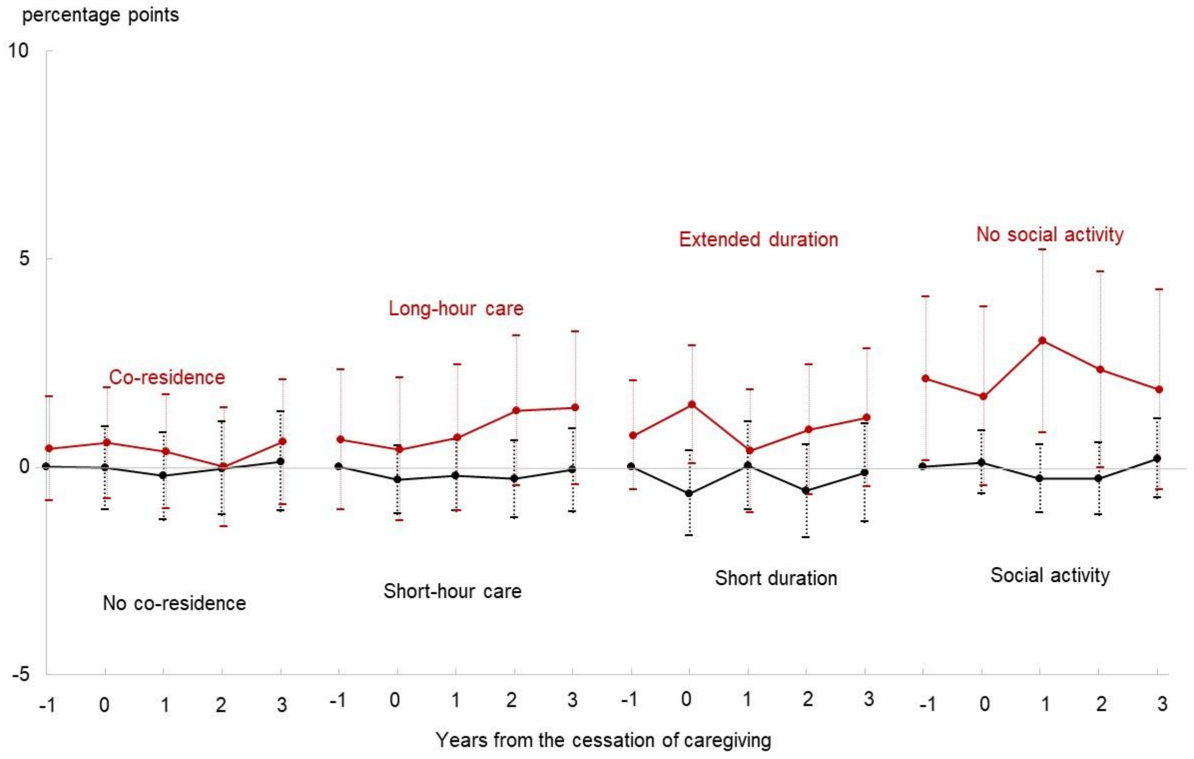

Supplement: Supplementary file 1 [file je-35-187-s001.pdf]
